# Dynamic evolution and spatial spillover effect of agricultural green development on eight economic regions in China

**DOI:** 10.1016/j.heliyon.2024.e33188

**Published:** 2024-06-17

**Authors:** Jingbo Shao, Lin Zhang, Chengzhi Cai

**Affiliations:** a Economic Institute, Guizhou University of Finance and Economics, Guiyang, 550025, China; b International Tourism Culture College, Guizhou Normal University, Guiyang, 550025, China

**Keywords:** Green development of agriculture, Economic regions, Dynamic evolution, Influencing factors, Spatial spillover effect

## Abstract

Promoting the green development of agriculture is of great significance to realize agricultural and rural modernization in China. Based on the existing research, this paper innovatively explores the dynamic and spatial effects of agricultural green development in the eight newly zoned regions of China's economy. Based on the panel data of 30 provinces in China from 2013 to 2022, this paper selects 20 indicators to measure the level of agricultural green development from five dimensions such as ecological protection, resource conservation, environment-friendly, green supply and economic growth by entropy weight method and uses non-parametric estimation method to analyze the dynamic evolution trend of agricultural green development in the whole country and its eight economic regions. Then, a spatial econometric model is constructed to further explore the influence mechanism and spatial spillover effect of each influencing factor on agricultural green development. The findings demonstrate that the level of agricultural green development in 30 provinces of China continuously improved during the study period, but the dynamic evolution trend characteristics in the whole country and its eight economic regions are not the same. Specifically, the development differences between the whole country, the northeast region, the eastern coast, the southern coast and the northwest region increased, while that between the northern coast, the Yellow River basin and the middle reaches of the Yangtze River first increased and then decreased, and that in the southwestern region gradually narrowed. There is a significant spatial spillover effect on agricultural green development and its influencing factors. Moreover, there is heterogeneity in the influence characteristics and spatial spillover effects of various influencing factors on agricultural green development among the eight economic regions. Therefore, it is proposed that eight economic regions in China should formulate differentiated development strategies, focus on educational and technological innovation etc., and further promote agricultural green development.

## 1 Introduction

The basic position of agriculture in China determines that the country must first strengthen the agricultural foundation if it is to be strong [[1], [2], [3]]. The green transformation of agriculture is not only the inherent requirement of high-quality agricultural development [4] but also the key link to promote the green transformation of the whole economy and society [2,5]. Since the reform and opening up, China's agriculture has achieved remarkable development achievements [6], and the agricultural production capacity and industrialization level have been steadily improved [7]. However, problems such as weak agricultural infrastructure, insufficient scientific and technological innovation capabilities [8], and agricultural non-point source pollution still exist [2,9]. In particular, the extensive use of chemical fertilizers and pesticides has brought a serious burden to the ecological environment [10,11]. The contradiction between economic development and ecological protection has become increasingly prominent [12,13]. The demand for green ecological transformation of agriculture is becoming more and more urgent [[14], [15], [16], [17]]. Based on this, the Chinese government has also introduced a series of policies. For example, in 2015, the Fifth Plenary Session of the Eighteenth Central Committee of the CPC put forward five development concepts [18], among which the “green development concept” gradually became the consensus of the whole party and the whole society, laying a good foundation for agricultural green development (AGD). In 2021, China's Ministry of Agriculture and Rural Affairs and other departments jointly issued the “14th Five-Year National Agricultural Green Development Plan” [19], listing the key tasks of AGD, requiring the same direction of goals, resources, and forces to promote the agriculture. In 2022, the No.1 Central Document proposed to promote AGD and rural areas, starting strengthening the comprehensive management of agricultural non-point source pollution and building a national pilot area of AGD [20], further, highlighting the importance and urgency of China's AGD. Therefore, it is particularly important to study the regional differences and spatial spillover effects of AGD in China.

In 2003, based on the nine principles of spatial proximity, similar natural conditions and similar resource endowment structure, the state divided mainland China into eight economic regions [21]. Based on this, this paper takes China and the eight major economic regions as the research area. Firstly, it measures the AGD from the perspective of harmonious coexistence between man and nature. Secondly, it further explores the dynamic evolution trend and spatial spillover effect of AGD in the eight economic regions. Finally, it analyzes the impact of various influencing factors on the AGD from both natural and social perspectives, to better sort out the differences in the AGD in different economic regions and their influencing mechanisms, and provide reference for improving the level of agricultural development and formulating differentiated policies.

The remaining part of this article is organized as follows. Section 2 reviews and comments on the literature, as well as combines the theoretical context, while Section 3 is for research methods, variables, and data, focusing on the construction of the index system, in Section 4 the dynamic evolution trend is analyzed, while in Section 5 the regression results are analyzed, and robustness tests and heterogeneity discussions are conducted. Finally Section 6 summarizes the research and puts forward countermeasures and suggestions.

## 2 Literature review and theoretical lenses

### 2.1 Literature review

The existing research results on AGD are relatively rich, which lays a solid foundation for this study. It mainly reflects the concept interpretation, index construction, attention area, and related influencing factors.

#### 2.1.1 Concept interpretation and index construction

The Green Revolution is often seen as the epitome of the dawn of technological progress and modernization in the agricultural sector of developing countries [22,23]. In a broad sense, green development is an ecological development under the concept and strategy of “Continual Development” [24,25]. On this basis, scholars have systematically studied the connotation of AGD. The AGD typically centers green agricultural products, leverages green technologies as a driving force, and relies on green policies as a safeguard, aiming to promote the coordinated development of agricultural ecological, social and economic benefits [26]. Some scholars believe that the AGD is to achieve green development in all aspects of agricultural production environment, production process and the quality of agricultural products [23,27]. Other scholars believe that China should follow the law of ecological development, make rational use of ecological resources, realize the self-recycling of resources, and emphasize the importance of ecological environment to the AGD [28]. In general, the scholars believe that the AGD is a development mode that focuses on agricultural resource conservation and rural environmental protection [29] and provides high-quality agricultural products to meet the growing needs of the people for a better life. Based on clarifying its connotation, the scholars have focused on the evaluation of AGD level. For the evaluation index system, the existing research mainly quantifies the dimensions of resource conservation, environmental friendliness, ecological conservation, economic growth and food security [30,31], analyzing the inter-provincial and annual AGD and the differences between various dimensions [32]. For evaluation methods, the entropy weight method [12], analytic hierarchy process [4] and linear weighting method [7] are often used to evaluate the level of AGD.

#### 2.1.2 Attention area and related influencing factors

Clarifying the spatial and temporal characteristics and driving factors of agricultural green GDP will help the spatial economic theory to explain the spatial agglomeration of economic factors [33]. For the concerned areas, the existing scholars mainly focus on the AGD in the provinces [1], Yangtze River Basin [34] and Yellow River Basin [35]. Using different analysis methods, some scholars found that the inter-provincial development of agricultural technology promotion is significantly varied [1,5,7,36,37]. For example, Deng et al. (2022) mainly used the spatial Dubin model to explore the spatial and temporal evolution characteristics and spatial spillover effects of agricultural green technology in China [36]. Chen et al. (2021) mainly used the spatial correlation network structure to explore inter-provincial spillover effects of AGD [5] and so on. However, many factors affect the AGD. For example, agricultural insurance is a common means to promote the AGD. It can not only encourage farmers to adopt green production technology and improve the production efficiency, but also achieve the purpose of reducing chemical input and protecting the environment [38]. The shape effectiveness of environmental Kuznets curve is also different between developed and developing groups [39,40]. From a policy perspective, it is recommended that governments formulate relevant policies to prioritize the efficiency of raw material resources in agricultural sector [41] and promote AGD. The impact of the integration of agricultural ecological efficiency can not be ignored [42,43]. It has a significant effect on promoting the sustainable development of agriculture [44] and further promotes the adjustment, optimization, transformation, and upgrade of agricultural industrial structure [45]. New agricultural cooperation [34], agricultural green technology innovation [46], regional exchange of green technology [27,47], appropriate subsidy policies [48] and digital economy [[49], [50], [51]] all have effectively promoted the AGD [52,53].

In general, present scholarly research on AGD mainly focuses on its measurement and influencing factors. Few scholars have analyzed the AGD in the eight economic regions of China from the perspective of harmonious coexistence between man and nature. In summary, compared with the existing research, the marginal contribution of the research is: (1) Based on the new perspective of harmonious coexistence between man and nature, this paper creatively constructs a five-dimensional index system of AGD level with ecological conservation, green supply, economic growth, resource conservation, and environmental friendliness. (2) Based on the index, this paper analyzes the dynamic evolution trend of AGD in China and its eight economic regions.

### 2.2 Theoretical lenses

Sustainable development is divided into weak and strong ones [6,54,55]. In 2015, China's “five new development concepts” was formally proposed at the Fifth Plenary Session of the 18th CPC Central Committee and the green development concept was included. Green development is the continuation of sustainable development, and so is sustainable development in China. In essence, it takes a strong sustainable path based on ecological economics. Both natural and non-natural capitals are irreplaceable, or at least some natural capital cannot be replaced. The complementary relationship between them means that both types of capital should be maintained and interdependent. The modernization of harmonious coexistence between man and nature is one of the main contents of Chinese modernization. How to solve the harmonious coexistence between man and nature is the main problem faced by green development [56]. The basic connotation of harmonious coexistence between man and nature is mainly elaborated from following three aspects. First of all, human beings and nature are the community of life. With the continuous progress of productivity, the ability of human beings to understand and transform nature has also increased [57]. Nature is the carrier of human survival and social development should be established in the concept of resource conservation and environment-friendly development [58]. Secondly, human beings and nature are the interests of the community. Natural ecosystem is a fundamental source of human interests and the natural basis for the sustainable development of generations. Human interests are based on natural ones. Green development can take into account both human and natural interests [59]. Thirdly, human beings and nature are the development community [60]. The development of human society should be coordinated with the proportion of the development of natural environment to achieve a harmonious coexistence between man and nature. Carding the theoretical context of AGD lays the foundation for the construction of subsequent relevant indicators.

**Fig. 1:**
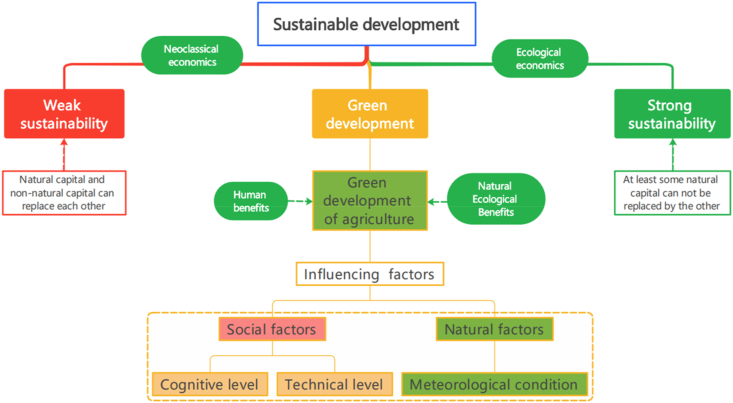
Theoretical context diagram and mechanism.

Combined with the above explanation, this paper analyzes the AGD from the perspectives of society and nature. On the one hand, from the perspective of social factors, the higher the level of people's awareness of green life is, the higher the willingness of people to adopt green production and lifestyle will be [61] and the education can improve their awareness of green development. The higher the level of information development is, the more they will have access to new things, and the easier it will be to form new life concepts [62]. The rising digital economy is providing inexhaustible impetus for green development and promoting the level of green development [63]. Advanced technology can improve agricultural production and resource utilization efficiency [64]. The initial investment in technology development may have a crowding-out effect on AGD. With effective application of technological promotion, the effect on AGD is becoming more and more obvious [65]. Therefore, this paper also introduces the square term of technology to examine the nonlinear relationship between technological level and AGD. On the other hand, from a natural point of view, the quality of meteorological conditions determines the yield of green agriculture to a certain extent. Accurate meteorological information can provide important guidance for farmers to sow the crop in time and adopt recommended agronomic practices accordingly [66]. The theoretical context and mechanism are shown in Fig. 1.

## 3 Research methods, variables and data

### 3.1 Research methods

#### 3.1.1 Entropy weight method

This paper follows the principles of representativeness, comparability, dynamics, and operability of index selection, screens AGD indicators, and establishes an index system. Due to the differences in the dimension, order of magnitude, and positive and negative orientation of each index, it is necessary to standardize the initial data. In the multi-index comprehensive evaluation, the determination of the weight of the evaluation index is related to the accuracy and credibility of empirical results. To avoid the deviation of subjective weight determination methods such as the expert consultation method, this paper uses the objective entropy weight method to calculate the index weight and calculates the comprehensive score of AGD based on the weight coefficient. Since the entropy weight method has been relatively mature, the specific details refer to the study of Tan and Qi (2023) [28].

#### 3.1.2 Non-parametric estimation method

In statistics, the parameter estimation method is often used to infer the distribution of population with sample data, which is the density function [67]. However, the parameter estimation has strict requirements for specific form of the population distribution [68]. If real population does not obey normal distribution, the parameter estimation method will produce large deviations. If the form of population distribution is not assumed, the time dynamic evolution trend of AGD level will be used to estimate the density function by non-parametric estimation method. Because the histogram is a discontinuous step function, this paper uses the kernel density estimation method to obtain smooth estimation of the density function [69]. The kernel density function is as formula (1).

(1) $$f\left(x\right)=\frac{1}{nh}\sum_{i=1}^{n}K\left[\frac{x_{i}-\bar{x}}{h}\right]$$

Where: $K\left[\frac{x_{i}-\bar{x}}{h}\right]$ is the kernel function, and the weight function in essence. h is the bandwidth, n is the number of samples, i is the province, $x_{i}$ is the observation value of each province, and $\bar{x}$ is the mean value. The larger the broadband value is, the larger both the neighborhood near by $\bar{x}$, and the deviation will be. However, the larger the broadband is, the smaller the variance var $\left[f\left(\bar{x}\right)\right]$ and the smoother the estimated density function will be. Therefore, it is particularly important to select appropriate optimal broadband. The functional relationship between optimal bandwidth and the sample size is as formula (2).

(2) $$h^{*}=1.3643\delta sn^{-0.2}$$

where δ is a constant and dependent on the kernel function in size, s is sample standard deviation, and n is sample observation value. The kernel function has a variety of forms respectively with different advantages. In this paper, Ivan Konnikov kernel or quadratic kernel is selected as shown in formula (3).

(3) $$K\left(x\right)=\frac{3}{4}\left(1-x^{2}\right)∙1\left(\left|x\right|<1\right)$$

Kernel density estimation generally observes the time trend of variables through a graph. The higher the peak is, the denser the data will be. The nuclear density curve moves to the right, indicating that the level of AGD is constantly improving, and vice versa. The lower the peak value and the wider the width are, the greater the difference in the level of AGD among the provinces will be, and vice versa. The multi-peak shape is obvious, indicating that there is a multi-polar differentiation phenomenon. The double peak transition to a single peak occurs, indicating that the phenomenon of polarization is weakening.

#### 3.1.3 Construction of the spatial econometric model

Before constructing the spatial model, the correlation test needs to be carried out first. In this paper, the global Moran index is used to measure the spatial correlation of AGD's levels. The specific details refer to the study of Hong et al. (2023) [14]. Spatial econometric model is mainly divided into the spatial autoregressive model or spatial lag model (SAR), spatial error model (SEM), and spatial Durbin model (SDM) [[70], [71], [72]]. When the model is set, the advanced LM test is needed, which needs to be judged by Spatial error and Spatial lag compared with the mixed OLS. If only the spatial error test results are significant, the spatial error model will be used. If only the spatial lag test results are significant, the spatial lag model will be selected. If both are significant, the robust LM test will be further performed. If the robust LM test is significant, the LR and Wald test will be performed. If the LR and Wald test is significant, the spatial Durbin model will be selected. Furthermore, the Hausman test is used to select the random utility model or the fixed effect model. If the p-value of the test result is significant, the null hypothesis will be rejected and the fixed effect model will be adopted. Otherwise, the random effect model is adopted.

The general models of spatial econometrics is as formula (4).

(4) $$y=\lambda W_{y}+X\beta +\mu$$

Where: *W* is the spatial weight matrix of *y*, *X* is the explanatory variable, and *μ* is the perturbation term.

### 3.2 Variable declaration

#### 3.2.1 Explained variable

Combined with the theoretical context of AGD, the index system of dependent variables is constructed from the five dimensions of resource conservation, environmental friendliness, ecological conservation, green supply, and economic growth. Resource conservation, environmental friendliness, and ecological conservation represent AGD at the level of natural ecological interests, while green supply and economic growth represent that at the level of human interests. The harmonious coexistence between man and nature requires a resource-saving and environment-friendly development path [[73], [74], [75]], which is the essential characteristic of the human and natural life, development community and the inherent attribute of AGD. Ecological conservation is helpful to improving the regional ecological environment and promoting the harmonious coexistence between man and nature [[76], [77], [78], [79]], which is the fundamental requirement of AGD. Under the background of food security, green supply means the level of agricultural products from the aspects of green output and food supply, which is the fundamental purpose of AGD [[80], [81], [82]]. Economic growth and environmental protection cannot be ignored, which is an important goal of AGD [[83], [84], [85]]. Therefore, from the perspective of economics as well as harmonious coexistence between man and nature, the level of AGD (represented by a score) is measured and shown in Table 1.

**Table 1:** Index system of AGD level.

Dimension	Secondary Index	Unit	Measure	Attribute	Weights
Economic growth	First industry added value of annual growth rate	%	First industry-added value/GDP	+	0.009
	Total agricultural output per unit of sown area	100 million yuan/thousand hectares	Gross agricultural output value/sown area	+	0.001
	Labor productivity	100 million yuan/ten thousand people	Total agricultural output/total agricultural population	+	0.023
	Proportion of agricultural financial support	%	Agricultural fiscal expenditure/total expenditure	+	0.122
Ecological conservation	Forest coverage	%	Forest coverage rate	+	0.001
	Soil erosion control acreage	Thousand hectares	Soil erosion control area	+	0.018
	Agricultural disaster resistance index	%	(Crop area-crop affected area)/Crop area	+	0.001
	Control rate of crop diseases, pests, weeds, and rodents	%	Crop pest control area/crop pest occurrence area	+	0.003
Resource- saving	Total power of agricultural machinery per unit of sown area	Ten thousand kilowatts/thousand hectares	Total power of agricultural machinery/total sown area	–	0.073
	Multiple Cropping Index of Cultivated Land		Sowing area/cultivated area	–	0.109
	Proportion of water-saving irrigation area	%	Water-saving irrigation area/total irrigation area	+	0.012
	Per unit water consumption of agricultural output value	Billion cubic meters/billion yuan	Agricultural water consumption/Total agricultural output value	–	0.094
Environmentally friendly	Intensity of agricultural fertilizer application	Thousand tons/thousand hectares	Agricultural fertilizer amount/sown area	–	0.061
	Use intensity of agricultural diesel oil	Thousand tons/thousand hectares	Agricultural diesel quantity/sown area	–	0.095
	Pesticide use intensity	Thousand tons/thousand hectares	Pesticide use/sown area negative	–	0.134
	Usage strength of agricultural plastic film	Thousand tons/thousand hectares	Agricultural film dosage/planting area	–	0.042
Green supply	Level of green food certifications	Piece	Number of green food certifications	+	0.146
	Level of Organic food certification	Piece	Organic food certification quantity	+	0.033
	Level of geographical indication certification of agricultural products	Piece	Number of geographical indication certification of agricultural products	+	0.017
	Grain product growth rate	%	(Grain yield in this year-that in previous year)/grain yield in previous year	+	0.006

#### 3.2.2 Related impact variables

On the one hand, this paper selects education level (‘edu’), information development level (‘inf’), and technical level (‘tec’) to represent social influencing factors. The level of education is expressed by the proportion of graduates from ordinary colleges and universities in each region accounting for the national totality. The level of information development is expressed by the proportion of the amount of regional post and telecommunication businesses accounting for the national totality. To investigate the nonlinear relationship of technical levels, this paper introduces the square term of technical level (‘tecc’). The technical level is expressed by the proportion of the number of domestic patent approved accounting for that applied. The reason why this paper uses the number of patent grants rather than that of applications is that there is a certain time lag effect in patents application. On the other hand, the meteorological (weather) conditions (‘wea’) are selected to represent the natural factors, which are represented by the proportion of the number of regional ecological and agricultural meteorological test stations accounting for the national totality.

### 3.3 Data sources and descriptive statistics

Based on the availability of data, the panel data of 30 provinces (municipalities and autonomous regions also called provinces except Hong Kong, Macao, Taiwan and Tibet) in China from 2013 to 2022 are selected as the sample. The required raw data are taken from *China Statistical Yearbook, China Rural Statistical Yearbook, China Agricultural Yearbook, Annual Report of Green Food Statistics* from 2014 to 2023 [[86], [87], [88], [89]], and the official authoritative data of provincial and municipal statistical bureaus. The linear interpolation method is used to process the missing data. Table 2 gives the descriptive statistics of each variable.

**Table 2:** Descriptive statistics of main indicators.

Variable	Measure	Mean	Std. Dev.	Min	Max
score	AGD	0.199	0.075	0.053	0.391
edu	Education level	0.033	0.015	0.004	0.064
inf	Level of information development	0.033	0.029	0.003	0.168
wea	Meteorological conditions	0.033	0.015	0.001	0.069
tec	Science and technology level	0.033	0.045	0.000	0.232
tecc	Square term of the technological level	0.003	0.008	0.000	0.054

Table 2 shows that the average value of AGD is overall low (0.199), ranging from a minimum of 0.053 to a maximum of 0.391 and there are some differences between those among the provinces. Compared with the mean values of other control variables, the difference in the development of technical level between regions is more obvious.

## 4 Dynamic evolution of AGD

### 4.1 Overall characteristics of AGD

The comprehensive scores and average rankings of AGD in each province of China during the period is shown in Table 3. It can be seen from Table 3 that the level of AGD in each province has increased year by year, and the AGD between the provinces has shown obvious differences. From 2013 to 2022, the average ranking of AGD in the top ten is Zhejiang, Shandong, Fujian, Heilongjiang, Sichuan, Yunnan, Hunan, Jiangxi, Jiangsu and Hubei. Among them, the comprehensive average scores of AGD in Zhejiang and Shandong were 0.320 and 0.313, respectively. In 2019, the AGD of Shandong Province showed a leap-forward development and then fell back, with a steady upward trend in general. The comprehensive average scores of AGD in Fujian and Heilongjiang were 0.276 and 0.267, respectively, showing the levels steadily improved. The comprehensive average scores of AGD in Sichuan and Yunnan were 0.266 and 0.265 respectively. The development law of these two provinces in 2020 was similar to that of Shandong in 2019. The comprehensive average scores of AGD in Hunan and Jiangxi were 0.260 and 0.259, respectively, with steadily improved levels. The comprehensive average scores of AGD in Jiangsu and Hubei were 0.254 and 0.252, respectively. In 2020, Jiangsu's AGD developed by leaps and bounds, and that of Hubei steadily improved. The last 10 provinces are Inner Mongolia, Tianjin, Qinghai, Ningxia, Shanxi, Xinjiang, Shanghai, Beijing, Jilin, and Chongqing. The comprehensive average score of Inner Mongolia's AGD was only 0.020 and relatively slow in general, with the level increasing slightly. The comprehensive average score of Tianjin's AGD was only 0.062, and almost showed a positive U-shaped development trend as a whole. Among those, the AGD in 2017–2018 was not ideal. The comprehensive average score of AGD in Qinghai was 0.080 and steadily improved year by year.

**Table 3:** Comprehensive scores of AGD in 30 provinces in China.

Province	Comprehensive score of AGD										Average ranking
	2013	2014	2015	2016	2017	2018	2019	2020	2021	2022	
Beijing	0.117	0.139	0.142	0.153	0.150	0.165	0.170	0.186	0.163	0.148	23
Tianjin	0.065	0.067	0.067	0.059	0.053	0.054	0.056	0.063	0.068	0.071	29
Hebei	0.191	0.173	0.171	0.182	0.175	0.170	0.194	0.189	0.198	0.202	17
Shanxi	0.089	0.094	0.102	0.105	0.106	0.108	0.117	0.143	0.167	0.186	26
Inner Mongolia	0.020	0.020	0.020	0.021	0.020	0.020	0.020	0.020	0.020	0.020	30
Liaoning	0.152	0.142	0.154	0.175	0.176	0.182	0.204	0.203	0.203	0.203	18
Jilin	0.149	0.134	0.133	0.144	0.146	0.157	0.164	0.165	0.180	0.188	22
Heilongjiang	0.183	0.182	0.203	0.222	0.230	0.267	0.324	0.311	0.355	0.391	4
Shanghai	0.104	0.117	0.121	0.116	0.119	0.106	0.133	0.168	0.153	0.138	24
Jiangsu	0.237	0.198	0.246	0.223	0.223	0.242	0.263	0.321	0.301	0.281	9
Zhejiang	0.272	0.294	0.321	0.324	0.331	0.328	0.341	0.325	0.329	0.336	1
Anhui	0.158	0.169	0.192	0.184	0.203	0.243	0.274	0.264	0.301	0.336	11
Fujian	0.218	0.244	0.254	0.258	0.259	0.265	0.280	0.306	0.326	0.347	3
Jiangxi	0.237	0.224	0.243	0.252	0.253	0.257	0.251	0.259	0.292	0.323	8
Shandong	0.262	0.289	0.299	0.272	0.289	0.300	0.379	0.334	0.346	0.360	2
Henan	0.142	0.144	0.152	0.177	0.169	0.194	0.220	0.223	0.249	0.269	15
Hubei	0.220	0.237	0.241	0.242	0.237	0.244	0.238	0.245	0.289	0.331	10
Hunan	0.195	0.200	0.206	0.216	0.221	0.356	0.248	0.293	0.322	0.347	7
Guangdong	0.170	0.181	0.189	0.183	0.186	0.194	0.188	0.207	0.222	0.236	14
Guangxi	0.156	0.159	0.171	0.172	0.178	0.183	0.184	0.200	0.240	0.282	16
Hainan	0.168	0.177	0.179	0.185	0.199	0.205	0.214	0.228	0.232	0.243	12
Chongqing	0.100	0.110	0.124	0.113	0.128	0.140	0.172	0.219	0.279	0.335	21
Sichuan	0.213	0.231	0.243	0.259	0.256	0.262	0.284	0.327	0.305	0.278	5
Guizhou	0.103	0.170	0.138	0.160	0.169	0.172	0.198	0.226	0.225	0.224	19
Yunnan	0.221	0.231	0.238	0.237	0.256	0.245	0.274	0.326	0.319	0.309	6
Shanxi	0.175	0.167	0.176	0.182	0.189	0.205	0.197	0.214	0.228	0.235	13
Gansu	0.130	0.133	0.140	0.159	0.150	0.168	0.203	0.208	0.231	0.254	20
Qinghai	0.065	0.067	0.069	0.071	0.080	0.078	0.079	0.094	0.097	0.096	28
Ningxia	0.089	0.086	0.098	0.085	0.088	0.100	0.090	0.091	0.110	0.122	27
Xinjiang	0.080	0.096	0.107	0.115	0.122	0.109	0.121	0.139	0.168	0.196	25

### 4.2 Time dynamic evolution trend

**Fig. 2:**
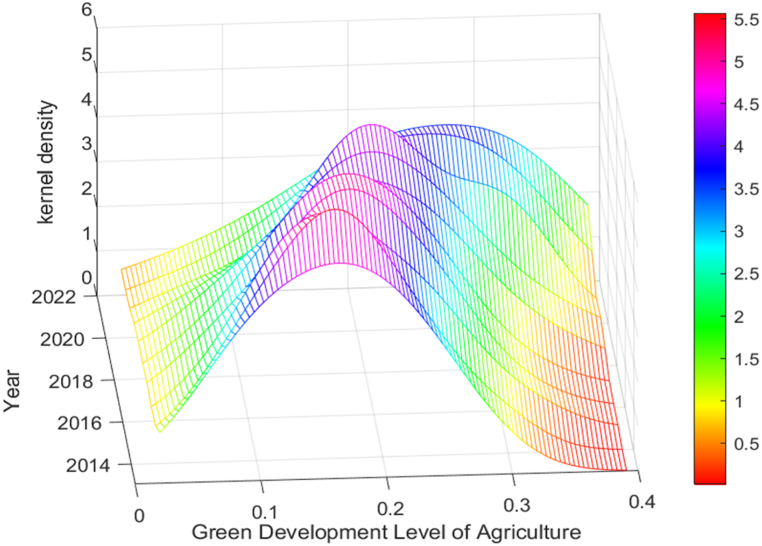
Kernel density curve of national AGD.

**Fig. 3:**
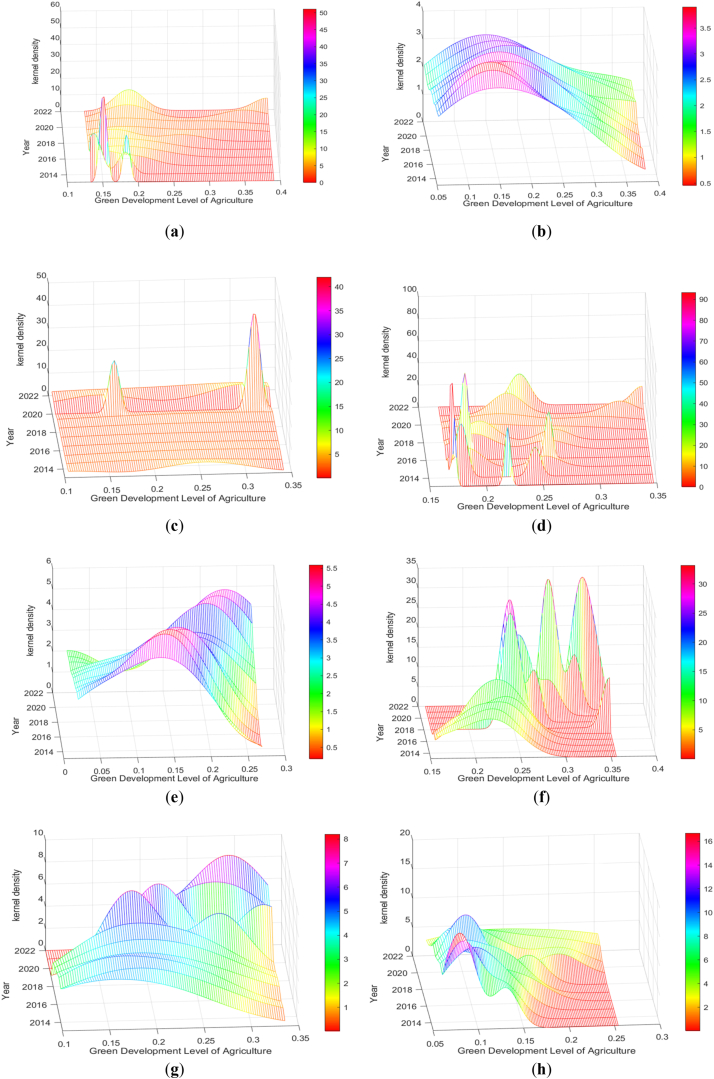
The kernel density curve of AGD in eight economic regions in China. (a) refers to the northeast region while (b) to the northern coastal region, (c) to the eastern coastal region, (d) to the southern coastal region, (e) to the middle reaches of the Yellow River, (f) to the middle reaches of the Yangtze River, (g) to the southwest region, and (h) to the northwest region, respectively.

This paper discusses the time-dynamic evolution trend of the whole country and eight major economic regions in China. The corresponding kernel density function trend chart is shown in Fig. 2, Fig. 3.

Taking 2013 as the base period, Fig. 2 shows that the density function center generally moves to the right, indicating that the level of AGD has been continuously improved. Specifically, the peak value from 2015 to 2017 remained unchanged, in 2018 decreased and remained unchanged until 2021, indicating no significant change in the inter-provincial differences between the periods of 2015–2017 and 2018 to 2021. The peak value decreased with the width increased in 2022, indicating that the inter-provincial differences in AGD expanded in 2022. Under the background of promoting the corresponding strategy, the AGD in some regions has achieved remarkable results.

In Fig. 3(a) the center of the density function moves to the right in Northeast China, the peak value decreases with the width increasing, indicating that the level of AGD has been significantly improved there, but the development differences between the provinces have increased. The possible explanation is that the northeast region is the main grain-producing area in China, where different AGD strategies adopted by various provinces lead to their differences. In Fig. 3(b) the center of the density function in the northern coastal area moves to the right and then to the left. The peak first decreased and then increased while the width increased and then decreased. It shows that the level of AGD in the northern coastal areas increases first and then decreases slightly and the development difference between the provinces widens first and then shrinks. Among them, the inter-provincial difference in 2018 is the largest. The possible explanation is that although both actively implement the AGD policy, Hebei and Shandong have a large agricultural volume whereas Beijing and Tianjin have a small one and the regional effects are significantly different. In Fig. 3 (c) the density function center of the eastern coastal area moves to the right as a whole, and the peak value rises slightly and then decreases. In 2020, a bimodal trend indicates that there is a polarization in the level of AGD in this region. In general, the level of AGD in the eastern coastal areas has increased year by year, and the inter-provincial differences have widened. The possible explanation is that Jiangsu and Zhejiang have relatively large volumes of agriculture and are conducive to large-scale production, resource utilization, and production efficiency, while Shanghai has a relatively small one and is relatively dependent on energy and related agricultural capital investment, so the gap in the level of AGD is widening in the region. In Fig. 3(d) the center of the density function of AGD moves to the right in the southern coastal areas, and the peak value decreases with the change range expanding, which indicates that the level of AGD has improved, with the inter-provincial development differences expanded. In Fig. 3(e) the center of the density function moves to the right in the middle reaches of the Yellow River, the peak value decreases first and then rises and the change interval expands first and then narrows, which indicates that the level of AGD in this region has improved, and the inter-provincial development gap has expanded first and then narrowed, tending to show a convergence state. In Fig. 3(f) the center of the density function generally moves to the right in the middle reaches of the Yangtze River, having the peak value first decreased and then rising with the change range first expanded and then shrunk, which indicates that the level of AGD has increased significantly in this region, but the inter-provincial development difference has expanded first and then narrowed. From 2018 to 2021, a right tail feature indicates that the level of AGD in the middle reaches of the Yangtze River was quite different at this time. In Fig. 3(g) the center of the density function in Southwest China moves to the right, the peak value increases and the width decreases, namely, the level of AGD in this region has been further improved, and the development differences between the provinces have been reduced. From 2019 to 2020, a bimodal trend indicates that there is polarization in the AGD at this time. In Fig. 3(h) the center of the density function moves to the right in the northwest region, the peak value decreases, and the change interval expands, indicating that the level of AGD has increased significantly and the inter-provincial development differences have expanded.

## 5 Spatial econometric regression results

### 5.1 Spatial correlation test

Before analyzing the spatial effect, the spatial correlation test needs to be carried out first. Table 4 conducts a global Moran index test on the level of AGD per year. The results showed that the AGD is significantly positively correlated, and the degree of agglomeration is obvious. The Moran index generally showed an upward trend, but there are some differences in the degree of spatial correlation. In addition to the Moran index test, this paper also uses the tests of the Geary index and the Getis & Ord index. The corresponding reference values of these three tests were 0.314, 0.557 and 0.017 respectively, and all passed the 1% significance test.^1^

**Table 4:** The Moran's index value of AGD in China from 2013 to 2022.

Variables	I	E(I)	Sd(I)	z	P-value
2013	0.183	−0.034	0.111	1.960	0.025
2014	0.248	−0.034	0.110	2.568	0.005
2015	0.282	−0.034	0.110	2.884	0.002
2016	0.200	−0.034	0.110	2.131	0.017
2017	0.243	−0.034	0.110	2.528	0.006
2018	0.237	−0.034	0.110	2.468	0.007
2019	0.204	−0.034	0.110	2.172	0.015
2020	0.262	−0.034	0.110	2.700	0.003
2021	0.290	−0.034	0.110	2.958	0.002
2022	0.310	−0.034	0.110	3.129	0.001

### 5.2 Analysis of influencing factors

Based on having spatial correlation, this paper has carried out spatial error maximum likelihood test, spatial lag maximum likelihood test, robust spatial error maximum likelihood test, robust spatial lag maximum likelihood test,^2^ and Hausman test (P = 0.4083). It can be seen that the spatial lag random effect model is suitable for this study.

Based on the above tests, this paper constructs the SAR model equation as shown in formula (2), in which the level of AGD (score) is the explained variable. Education level, information development level, meteorological conditions, technical level, and its square terms are used as explanatory variables.

(5) $${score}_{it}=\beta_{0}+\rho \sum_{j}^{n}W_{ij}{agr}_{it}+\beta_{1}{edu}_{it}+\beta_{2}\;\inf_{it}+\beta_{3}{wea}_{it}+\beta_{4}{tec}_{it}+\beta_{5}{tecc}_{it}+\varepsilon_{it}$$

Where: i and j represent different provinces, t represents different years within the study period, $W_{ij}$ is the spatial weight matrix, the relevant letters before each explanatory variable are the regression coefficients, and $\varepsilon_{it}$ is the random error term.

The regression results of the equation are shown in Table 5 in which Columns (1) and (2) are ordinary least squares methods and stepwise regression of the square term of technical level is added in turn. The results show that education level, meteorological conditions, and technical level have a significant impact on the dependent variable. The coefficient scores are 1.509, 1.027, 1.733, and −6.741 respectively, indicating that there is an inverted U-shaped relationship between technical level and AGD level. Columns (3) and (4) are the square fixed effect model regression of SAR in turn adding technology. The results show that the level of information development and that of technology have significant effects, with coefficients of 1.078, −1.556, and 5.143, respectively, indicating that there is a positive U-shaped relationship between the level of technology and that of AGD. Columns (5) and (6) are the random effect regression results of the square term of SAR in turn. The results show that education level, information level, meteorological conditions, and technical level all have a significant impact on the level of AGD, with their coefficients of 1.660, 0.861, 1.352, −1.421 and 4.624, respectively, indicating that various influencing factors have an important impact on AGD. There is a positive U-shaped relationship between technical level and AGD level, and the effect is significant. The possible explanation is that the development of initial technical level has a certain crowding-out effect on AGD. However, the improvement and progress of technology have significantly promoted the AGD and improved its level. This is consistent with the results of Guo et al. (2021), Zhao et al. (2021) and Chi et al. (2021) who emphasized the impacts of education [90], informatization level [91] and technology [92] on AGD, respectively. On this basis, this paper comprehensively examines various influencing factors such as education, information level, meteorological conditions, scientific and technological level, and emphasizes dynamic evolution trend and spatial effect, which provides a more scientific empirical reference for the formulation of AGD policy. In addition, the results of Table 5 also showed that ignoring the nonlinear relationship between spatial spillover effect and technical level and AGD will lead to large errors in the estimation. This is similar to the results of Chen et al. (2022) and Lei et al. (2024) that the spatial spillover effect of AGD between the provinces is obvious [5] and that there is a nonlinear relationship between the digitization and green development [93].

**Table 5:** Regression results of different models.

Variables	(1)	(2)	(3)	(4)	(5)	(6)
edu	2.261***	1.509***	0.877	1.208	0.957	1.660**
	(0.362)	(0.416)	(1.183)	(1.162)	(0.748)	(0.784)
inf	−0.142	−0.462	0.884***	1.078***	0.506*	0.861***
	(0.289)	(0.298)	(0.322)	(0.321)	(0.284)	(0.298)
wea	0.770**	1.027***	0.748	0.665	1.703***	1.352**
	(0.302)	(0.306)	(1.402)	(1.373)	(0.642)	(0.663)
tec	0.124	1.733***	0.0474	−1.556***	−0.0500	−1.421***
	(0.177)	(0.494)	(0.132)	(0.464)	(0.125)	(0.430)
tecc		−6.741***		5.143***		4.624***
		(1.937)		(1.432)		(1.387)
_cons	0.070***	0.084***			−0.066**	−0.064**
	(0.015)	(0.016)			(0.029)	(0.029)
Spatial						
rho			0.447***	0.447***	0.481***	0.459***
			(0.060)	(0.059)	(0.056)	(0.056)

Note: The markers *, * * and * * * are significant levels of 10 %, 5 % and 1 %, respectively (the same is in the following tables).

### 5.3 Robustness test

In addition to constructing the adjacency space weight matrix, this paper also constructs the inverse distance space weight matrix, the nested space weight matrix, and the economic geographic space weight matrix, which are respectively substituted into the benchmark model equations set. Through sequential regression, it is found that no matter which spatial weight matrix is based on, the regression results are significant and so are the characteristics. It can be seen that this paper uses the adjacency weight matrix for spatial lag model regression, and gains robust results. The regression results of different weights are shown in Table 6.

**Table 6:** Regression results of SAR random effects model with different weights.

Variables	(1)		(2)		(3)		(4)	
	Coefficient	P-value	Coefficient	P-value	Coefficient	P-value	Coefficient	P-value
edu	1.660**	0.034	2.050**	0.023	1.922**	0.034	2.259**	0.011
inf	0.861***	0.004	1.353***	0.000	1.332***	0.000	1.313***	0.000
wea	1.352**	0.041	1.541*	0.050	1.624**	0.038	1.290*	0.093
tec	−1.421***	0.001	−1.899***	0.000	−1.896***	0.000	−1.784***	0.000
tecc	4.624***	0.001	6.179***	0.000	6.280***	0.000	5.738***	0.000

Note: Regressions of (1), (2), (3), (4) are regression results based on adjacency spatial weight matrix, inverse distance spatial weight matrix, nested spatial weight matrix, and economic geography matrix respectively.

### 5.4 Decomposition of spatial spillover effect

Based on the spatial auto-regressive results, the spatial spillover effect is further analyzed and decomposed. The results of the spatial lag equation are further unbiased by partial differential method, and the spatial effect is decomposed into direct and indirect ones. The results are shown in Table 7. The direct and indirect effects of education level are significantly positive, with coefficients of 1.775 and 1.375, respectively, indicating that the level of local education not only has a significant positive impact on the AGD in the same region but also exerts significantly an impact on that in neighboring regions. The direct and indirect effects of the information level are significantly positive at a 1 % significant level, with coefficients of 0.885 and 0.667 respectively, indicating that the information level has a very significant impact on the AGD in the same region and adjacent ones. Although the rural information network is relatively underdeveloped in China, and the acceptance ability of left-behind middle-aged and elderly farmers using the information network is also limited, with the further improvement of the information level, the effect of AGD will be more significant. The direct and indirect effects of meteorological conditions on the AGD are significantly positive, with coefficients of 1.497 and 1.141, respectively, indicating that the proportion of local ecological and agrometeorological test sites accounting for national totality has a significant impact on the AGD in the same region and adjacent regions. The direct and indirect effects of the first term of the technical level are significantly negative with their coefficients of −1.460 and −1.118 respectively, while those of the second term are significantly positive with their coefficients of 4.773 and 3.656 respectively. It showed that the technical level has a significant positive U-shaped relationship with the AGD not only in the same region but also in the neighbors. This is similar to the research results of Xiao et al. (2022) and Cheng et al. (2023) that the spillover effect of technological progress has a non-linear contribution to Green Total Factor Productivity [11] and that the direct impact of technological level on AGD is greater than the indirect impact [1], respectively. In the early stage of technological development, it is necessary to invest in production factors such as capital, which has a certain crowding-out effect on the AGD. However, once the technological achievements are put into operation, technological development will have a very significant positive effect on the AGD in the same region and adjacent regions [36].

**Table 7:** Spatial effect decomposition of AGD.

Variables	Direct effect		Indirect effect		Total effect	
	Coefficient	P-value	Coefficient	P-value	Coefficient	P-value
edu	1.775**	0.035	1.375*	0.066	3.150**	0.042
inf	0.885***	0.001	0.667***	0.004	1.552***	0.001
wea	1.497**	0.039	1.141*	0.054	2.638**	0.040
tec	−1.460***	0.002	−1.118**	0.012	−2.579***	0.003
tecc	4.773***	0.001	3.656**	0.011	8.430***	0.003

### 5.5 Heterogeneity analysis

From the above analysis, it is known that there is a significant spatial spillover effect in AGD. To test whether the spatial effect of AGD is heterogeneous among eight economic regions in China, this paper constructs the spatial weight matrix and the spatial econometric regression (Table 8). The columns of (M1) to (M8) of Table 8 are the corresponding spatial regression results of the northeast region, the northern coast, the eastern coast, the southern coast, the middle reaches of the Yellow River, the middle reaches of the Yangtze River, the southwest region and the northwest regions, respectively. The main effect regression results showed that the effect of education level in the eight economic regions is significant, but the effects are significantly negative in the northern and southern coastal areas. The possible explanation is that the level of education is often restricted by the level of economic development, and the early stage of economic development has a certain crowding-out effect on green development [94]. The level of information development has a significant impact on the northeast region, the eastern coast, the southern coast, the southwest, and the northwest regions and the impact coefficients are significantly negative in the northeast and eastern coastal regions. The possible explanation is that the phased improvement of the level of information development also has a crowding-out effect on the AGD. The meteorological conditions have a significant impact respectively on the northeast region, the middle reaches of the Yellow River, the middle reaches of the Yangtze River, the southwest region, and the northwest region. According to the statistical data, the overall development of meteorological conditions is not good, but that in the northeast region is relatively good, which has a significant role in promoting the AGD. The technical level and its square term have significant effects on the northern coast, the eastern coast, the middle reaches of the Yellow River, and the northwest region, and the effects of the northern coast and the middle reaches of the Yellow River showed an inverted “U” relationship. The industrial economies of Beijing, Tianjin, Hebei, Shandong, and Henan are prominent, and the improvement of the technical level at this stage further promotes the development of the industrial economy and weakens the AGD. This is similar to the research results of Chen et al. (2021), Guo et al. (2023) and Liu et al. (2020) that the AGD is generally growing with a significant regional heterogeneity [7], and that the overall level of AGD is not high with obvious growth differences between the provinces [1,4]. This paper focuses on the analysis of the heterogeneity of AGD in the eight economic regions of China, and aims to provide corresponding policy guidance.

**Table 8:** Spatial regression and spatial effect decomposition of AGD in eight economic regions in China.

	M1	M2	M3	M4	M5	M6	M7	M8
Main								
edu	5.268***	−7.659***	16.68***	−8.000***	6.451*	3.049***	4.167**	7.879***
	(1.578)	(2.049)	(5.166)	(0.818)	(3.355)	(0.822)	(1.917)	(1.410)
inf	−2.862***	−0.0327	−3.421**	2.049***	−0.0168	−1.296	2.893**	3.060***
	(0.564)	(1.006)	(1.743)	(0.431)	(1.278)	(0.829)	(1.281)	(0.890)
wea	2.175*	1.607	−2.840	0.187	−5.446***	−2.155**	−0.400	−1.690***
	(1.129)	(1.316)	(1.939)	(2.521)	(1.742)	(0.847)	(1.132)	(0.478)
tec	1.505	30.180***	−4.869**	−0.930	4.320*	1.554	−5.894*	−34.380***
	(1.355)	(5.947)	(2.218)	(0.863)	(2.442)	(1.694)	(3.117)	(7.565)
tecc	10.80	−838.9***	12.87**	7.088*	−59.16*	−5.428	94.99	5668.8***
	(13.220)	(181.100)	(6.197)	(3.658)	(32.300)	(5.420)	(70.20)	(905.8)
_cons	−0.0701***	0.0959	−0.0975**	0.334**	0.00940	0.0911***	−0.0592	0.0225
	(0.0210)	(0.0757)	(0.0440)	(0.139)	(0.104)	(0.0273)	(0.0416)	(0.0140)
Spatial								
lambda	0.442***							
	(0.133)							
rho		0.423***	0.448***	0.678***	0.316**	0.505***	0.618***	0.449***
		(0.111)	(0.146)	(0.0628)	(0.160)	(0.117)	(0.0859)	(0.0622)
Direct								
edu		−8.200***	19.440***	−12.700***	6.982*	3.945***	5.241**	8.919***
		(2.178)	(5.875)	(2.535)	(3.834)	(1.197)	(2.419)	(1.655)
inf		−0.129	−4.058**	3.194***	−0.128	−1.748*	3.463**	3.355***
		(0.934)	(1.630)	(0.826)	(1.181)	(0.995)	(1.366)	(0.860)
wea		1.842	−3.218	0.375	−5.689***	−2.748**	−0.314	−1.851***
		(1.566)	(2.174)	(4.321)	(1.886)	(1.133)	(1.369)	(0.502)
tec		32.62***	−5.557**	−1.360	4.511	2.176	−7.025*	−38.83***
		(6.135)	(2.621)	(1.529)	(2.840)	(2.137)	(4.067)	(10.440)
tecc		−914.1***	14.53**	10.71	−61.79*	−7.702	106.3	6343.6***
		(190.9)	(7.364)	(6.911)	(36.390)	(7.003)	(90.320)	(1272.600)
Indirect								
edu		−3.304**	11.09*	−12.61***	3.311	2.611*	5.899*	5.581***
		(1.416)	(6.622)	(4.343)	(3.537)	(1.525)	(3.288)	(1.626)
inf		−0.0131	−2.128*	3.171***	0.0521	−1.134	3.862**	2.089***
		(0.415)	(1.101)	(1.224)	(0.658)	(0.836)	(1.783)	(0.680)
wea		0.756	−1.686	0.387	−2.521	−1.836	−0.266	−1.135***
		(0.737)	(1.565)	(4.351)	(2.240)	(1.230)	(1.652)	(0.325)
tec		12.86***	−3.291	−1.417	1.509	1.452	−8.130	−24.86**
		(3.906)	(2.585)	(1.629)	(1.396)	(1.676)	(5.390)	(9.917)
tecc		−361.100***	8.736	11.010	−23.240	−5.242	124.500	4027.2***
		(116.500)	(7.229)	(8.078)	(23.190)	(5.609)	(114.000)	(1345.900)
Total								
edu		−11.500***	30.530***	−25.310***	10.290	6.557**	11.140**	14.500***
		(3.255)	(11.270)	(6.840)	(6.974)	(2.598)	(5.542)	(3.115)
inf		−0.142	−6.186**	6.364***	−0.0764	−2.882	7.325**	5.444***
		(1.336)	(2.421)	(2.026)	(1.778)	(1.772)	(3.045)	(1.481)
wea		2.598	−4.904	0.762	−8.210**	−4.584**	−0.580	−2.986***
		(2.261)	(3.559)	(8.652)	(3.808)	(2.277)	(3.006)	(0.788)
tec		45.48***	−8.848*	−2.776	6.020	3.628	−15.160	−63.690***
		(7.819)	(4.934)	(3.146)	(3.725)	(3.742)	(9.282)	(20.000)
tecc		−1275.300***	23.270*	21.710	−85.030	−12.940	230.800	10370.800***
		(251.100)	(13.940)	(14.910)	(52.570)	(12.380)	(202.100)	(2554.600)
N	30	40	30	30	40	40	50	40

Note: Standard errors are in the parentheses. After model screening, the spatial error random effect model is suitable for Northeast China whereas the spatial lag random effect model for the other regions.

The regression results of spatial spillover effect showed that the indirect effect of education level in the middle reaches of the Yellow River is not significant, indicating that improving education level in the same region will not promote the improvement of AGD in adjacent regions. The level of information in the eastern coastal, southern coastal, southwestern, and northwestern regions significantly affects the level of AGD not only in the same region but also in neighboring regions. However, the level of information development in the middle reaches of the Yangtze River has no significant effect on that in neighboring regions. The meteorological conditions have significant direct and indirect effects on AGD in the northwest region. But in the middle reaches of the Yellow River and the middle reaches of the Yangtze River, there is only a certain crowding out effect of meteorological conditions on AGD only in the same region. The technical level and its square term have significant direct and indirect effects on AGD in the northern coastal and northwestern regions. But in the eastern coastal areas, there is only a significant positive “U” relationship between meteorological condition and AGD in the same region and no significant indirect spillover effect on adjacent regions.

## 6 Conclusions and policy suggestions

### 6.1 Conclusions

Based on the panel data of 30 provinces in China from 2013 to 2022, this paper first establishes a comprehensive index system to evaluate the level of AGD from the perspective of harmonious coexistence between man and nature and explores the time-dynamic evolution trend of AGD level in the whole country and the eight economic regions through kernel density analysis. Then, the spatial spillover effect and the mechanism of influencing factors are analyzed by constructing a spatial econometric model. On this basis, the heterogeneity of the eight economic regions is discussed. The following conclusions are drawn as follows. (1) From 2013 to 2022, the level of AGD in 30 provinces in China has been continuously improved. The dynamic evolution trend characteristics of AGD in the whole country and the eight economic regions are different. Among them, the development differences between the whole country, the northeast, the eastern coast, the southern coast and the northwest regions have increased; the development differences between the northern coast, the Yellow River and the middle reaches of the Yangtze River increased first and then decreased. The difference in development in the southwest region has gradually narrowed. (2) There is a significant spatial spillover effect, and a heterogeneity in the influence characteristics and spatial spillover effects of various influencing factors on AGD among the eight economic regions. (3) On the whole, the level of technology has a significant positive U-shaped impact on the level of AGD. Education level, information development level, and meteorological conditions all have a significant positive impact on the level of AGD. This study systematically examines distributive dynamics, dynamic evolution trend, and related influence mechanism of AGD level through scientific methods, tests the robustness of the results, and provides reference for improving green development of agriculture and formulating differentiated policies in China.

### 6.2 Policy suggestions

Based on the above research conclusions, the following policy implications are obtained. Firstly, China should promote scientific and technological innovation and achievement transformation. It is necessary to strengthen cooperation with agricultural research institutions and universities, introduce and promote advanced agricultural technology and equipment, and improve the efficiency and quality of agricultural production. At the same time, it is also necessary to strengthen agricultural science and technology innovation and achievement transformation, and promote the upgrade and transformation of agricultural industry. Secondly, the education is an important factor affecting the AGD and should be focused on. Local governments should carry out relevant education training, improve the development cognition of agricultural producers, train agricultural technicians to develop agricultural production technology, and guide the consumers' consumption preference for green food and organic food. Thirdly, China should strengthen policy support and financial services. The government should introduce relevant policies to support and guide the AGD. For example, they should provide financial subsidies, tax incentives and other policy measures to reduce the cost of agricultural green production. At the same time, financial service innovation should be strengthened to provide financing support for green agricultural enterprises and promote AGD. Fourthly, China should implement a differentiated AGD strategy. Based on regional characteristics and advantages, the fine agriculture and characteristic industries should be vigorously developed. For example, agricultural producers can cultivate varieties of products with local characteristics, improve product quality and build brand influence. At the same time, it can also develop characteristic aquaculture, leisure agriculture, etc., and enrich the agricultural industrial structure. Last but not the least, China should strengthen the cooperation and exchanges with international organizations and other countries, learn from international advanced experience, introduce foreign advanced green agricultural technology and management mode, and further promote the AGD nationwide.

### 6.3 Limitations and future research

Although this paper systematically sorts out the differences, dynamic evolution trends and spatial spillover effects of agricultural green development in the whole country and eight economic regions of China, there are still some deficiencies. In the next study, we can potentially extend following two aspects:

Firstly, the micro-level statistical data of agricultural green development is difficult to obtain completely, which leads to the lack of research on the cities of prefecture-level. The next step is to try to use field research to obtain micro-data for the study of agricultural green development in the counties or towns.

Secondly, the index system of agricultural green development level still needs being improved. The imperfection of index system leads to the decrease of the accuracy of the calculation results. The next step will be to improve the construction dimension of agricultural green development index system to ensure that the measurement results are more accurate and reliable.

## Data availability statement

Data will be made available on request.

## CRediT authorship contribution statement

**Jingbo Shao:** Writing – original draft, Validation, Resources, Methodology, Investigation, Funding acquisition, Formal analysis, Data curation, Conceptualization. **Lin Zhang:** Investigation, Formal analysis. **Chengzhi Cai:** Writing – review & editing, Supervision.

## Declaration of competing interest

The authors declare that they have no known competing financial interests or personal relationships that could have appeared to influence the work reported in this paper.

## Figures and Tables

**Table A:** Spatial correlation test of AGD.

Moran's I	Variable	I	E(I)	Sd(I)	z	P-value
	score	0.314	−0.003	0.039	8.219	0.000
**Geary's c**	**Variable**	**c**	**E(c)**	**Sd(c)**	**z**	**P-value**
	score	0.557	1.000	0.049	−9.009	0.000
**Getis & Ord's G**	**Variable**	**G**	**E(G)**	**Sd(G)**	**z**	**P-value**
	score	0.017	0.015	0.000	7.212	0.000

**Table B:** LM and robust LM test regression results.

Test		Statistic	P-value
Spatial error:	Lagrange multiplier	33.594	0.000
	Robust Lagrange multiplier	1.815	0.178
Spatial lag:	Lagrange multiplier	48.026	0.000
	Robust Lagrange multiplier	16.247	0.000

## Footnotes

1 Table A in the appendix is the result of the spatial correlation test of AGD with Moran, Geary and Getis & Ord, respectively.

2 Table B in the appendix is the regression results of LM and robust LM tests.

## References

[1] Guo F., Cheng L., Gao Y., Dai X. (2023). Spatio-temporal comprehensive measurement of China's agricultural green development level and associated influencing factors. PLoS One.

[2] Hou D., Wang X. (2022). Measurement of agricultural green development level in the three provinces of northeast China under the background of rural vitalization strategy. Front. Public Health.

[3] Zhang Y.-f., Ji M.-x., Zheng X.-z. (2023). Digital economy, agricultural technology innovation, and agricultural green total factor productivity. Sage Open.

[4] Liu Y., Sun D., Wang H., Wang X., Yu G., Zhao X. (2020). An evaluation of China's agricultural green production: 1978–2017. J. Clean. Prod..

[5] Chen Z., Sarkar A., Rahman A., Li X., Xia X. (2022). Exploring the drivers of green agricultural development (GAD) in China: a spatial association network structure approaches. Land Use Pol..

[6] Sneddon C., Howarth R.B., Norgaard R.B. (2006). Sustainable development in a post-Brundtland world. Ecol. Econ..

[7] Chen Z., Li X., Xia X. (2021). Measurement and spatial convergence analysis of China's agricultural green development index. Environ. Sci. Pollut. Res..

[8] Zhang F., Wang F., Hao R., Wu L. (2022). Agricultural science and technology innovation, spatial spillover and agricultural green development—taking 30 provinces in China as the research object. Appl. Sci..

[9] Liu D., Zhu X., Wang Y. (2021). China's agricultural green total factor productivity based on carbon emission: an analysis of evolution trend and influencing factors. J. Clean. Prod..

[10] Lehtonen H., Rämö J. (2022). Development towards low carbon and sustainable agriculture in Finland is possible with moderate changes in land use and diets. Sustain. Sci..

[11] Xiao S., He Z., Zhang W., Qin X. (2022). The agricultural green production following the technological progress: evidence from China. Int. J. Environ. Res. Publ. Health.

[12] Guo H., Xu S., Pan C. (2020). Measurement of the spatial complexity and its influencing factors of agricultural green development in China. Sustainability.

[13] Guo H., Gu F., Peng Y., Deng X., Guo L. (2022). Does digital inclusive finance effectively promote agricultural green development?—a case study of China. Int. J. Environ. Res. Publ. Health.

[14] Hong M., Tian M., Wang J. (2023).

[15] Wang Q., Wang K. (2022). The synergistic and trade-off effects of economic-environmental-health improvement in agriculture sector: evidence from China. Environ. Sci. Pollut. Res..

[16] Wang W., Li K., Liu Y., Lian J., Hong S. (2022). A system dynamics model analysis for policy impacts on green agriculture development: a case of the Sichuan Tibetan Area. J. Clean. Prod..

[17] Xu L.-Y., Jiang J., Du J.-G. (2023). How do environmental regulations and financial support for agriculture affect agricultural green development? The mediating role of agricultural infrastructure. J. Environ. Plann. Manag..

[18] Central Committee of the CPC (2015). The full text of the communiqué of the Fifth plenary session of the 18^th^ central committee of the communist party of China. https://china.huanqiu.com/article/9CaKrnJR1eH.

[19] (2021). National Agricultural Green Development Plan.

[20] Ministry of Agriculture and Rural Affairs of the People’s Republic of China (2022). No.1 Document of the Central Committee of the Communist Party of China.

[21] Niu X., Ma Z., Ma W., Yang J., Mao T. (2024). The spatial spillover effects and equity of carbon emissions of digital economy in China. J. Clean. Prod..

[22] Jänicke M. (2012). “Green growth”: from a growing eco-industry to economic sustainability. Energy Pol..

[23] Koohafkan P., Altieri M.A., Gimenez E.H. (2012). Green agriculture: foundations for biodiverse, resilient and productive agricultural systems. Int. J. Agric. Sustain..

[24] Cabral L., Pandey P., Xu X. (2021). Epic narratives of the green revolution in Brazil, China, and India. Agric. Hum. Val..

[25] van Etten J. (2022). Revisiting the adequacy of the economic policy narrative underpinning the green revolution. Agric. Hum. Val..

[26] Li M., Wang J., Zhao P., Chen K., Wu L. (2020). Factors affecting the willingness of agricultural green production from the perspective of farmers' perceptions. Sci. Total Environ..

[27] Adnan N., Nordin S.M., Bahruddin M.A., Tareq A.H. (2019). A state-of-the-art review on facilitating sustainable agriculture through green fertilizer technology adoption: assessing farmers behavior. Trends Food Sci. Technol..

[28] Tan H., Qi X. (2023). Synergistic interconstruction of the green development concept in Chinese rural ecological agriculture. Sustainability.

[29] Shen J., Zhu Q., Jiao X., Ying H., Wang H., Wen X., Xu W., Li T., Cong W., Liu X. (2020). Agriculture green development: a model for China and the world. Frontiers of Agricultural Science Engineering and Technology.

[30] Tao Z., Xiang G. (2022). Empirical measurement and evaluation of rural green development: take Hunan Province, China as an example. Environ. Earth Sci..

[31] Gu R., Duo L., Guo X., Zou Z., Zhao D. (2023). Spatiotemporal heterogeneity between agricultural carbon emission efficiency and food security in Henan, China. Environ. Sci. Pollut. Res..

[32] Xu B., Chen W., Zhang G., Wang J., Ping W., Luo L., Chen J. (2020). How to achieve green growth in China's agricultural sector. J. Clean. Prod..

[33] Li Z., Jin M., Cheng J. (2021). Economic growth of green agriculture and its influencing factors in China: based on emergy theory and spatial econometric model. Environ. Dev. Sustain..

[34] Cui X., Cai T., Deng W., Zheng R., Jiang Y., Bao H. (2022). Indicators for evaluating high-quality agricultural development: empirical study from Yangtze River economic belt, China, social indic. Res..

[35] Chen S., Wang X., Yao S. (2023). National water-saving city and its impact on agricultural total factor productivity: a case study of nine provinces along the Yellow River, China. J. Clean. Prod..

[36] Deng Y., Cui Y., Khan S.U., Zhao M., Lu Q. (2022). The spatiotemporal dynamic and spatial spillover effect of agricultural green technological progress in China. Environ. Sci. Pollut. Res..

[37] Xu B., Niu Y., Zhang Y., Chen Z., Zhang L. (2022). China's agricultural non-point source pollution and green growth: interaction and spatial spillover. Environ. Sci. Pollut. Res..

[38] Li H., Tang M., Cao A., Guo L. (2022). Assessing the relationship between air pollution, agricultural insurance, and agricultural green total factor productivity: evidence from China. Environ. Sci. Pollut. Res..

[39] Mellaku M.T., Sebsibe A.S. (2022). Potential of mathematical model-based decision making to promote sustainable performance of agriculture in developing countries: a review article. Heliyon.

[40] Bergius M., Benjaminsen T.A., Maganga F., Buhaug H. (2020). Green economy, degradation narratives, and land-use conflicts in Tanzania. World Dev..

[41] Li C., Shi Y., Khan S.U., Zhao M. (2021). Research on the impact of agricultural green production on farmers' technical efficiency: evidence from China. Environ. Sci. Pollut. Res..

[42] Iannucci G., Martellozzo F., Randelli F. (2022). Sustainable development of rural areas: a dynamic model in between tourism exploitation and landscape decline. J. Evol. Econ..

[43] Sun B., Wang G., Liu Y. (2023). Leisure agriculture and rural tourism benefit analysis on eco-environmental resource use. Sustainability.

[44] Wang J., Zhou F., Xie A., Shi J. (2022). Impacts of the integral development of agriculture and tourism on agricultural eco-efficiency: a case study of two river basins in China, Environment. Development and Sustainability.

[45] Liu F., Wang C., Yingyan Z., Zhou S., Yaliu Y., Wu X., Fagang H., Conghu L. (2022). Correction to: data-driven analysis and evaluation of regional agriculture for high quality development of Anhui Province in the Yangtze River Delta. Environ. Sci. Pollut. Res..

[46] He W., Li E., Cui Z. (2021). Evaluation and influence factor of green efficiency of China's agricultural innovation from the perspective of technical transformation. Chin. Geogr. Sci..

[47] Cui Z., Wang F. (2023). The spatiotemporal dynamic and spatial spillover effect of green finance efficiency in China: analysis based on super-SBM model and spatial Durbin model. Environ. Sci. Pollut. Res..

[48] Luo J., Huang M., Bai Y. (2023). Promoting green development of agriculture based on low-carbon policies and green preferences: an evolutionary game analysis. Environ. Dev. Sustain..

[49] Shen Z., Wang S., Boussemart J.-P., Hao Y. (2022). Digital transition and green growth in Chinese agriculture. Technol. Forecast. Soc. Change.

[50] Zhou X., Chen T., Zhang B. (2023). Research on the impact of digital agriculture development on agricultural green total factor productivity. Land.

[51] Hong M., Tian M., Wang J. (2022). Digital inclusive finance, agricultural industrial structure optimization and agricultural green total factor productivity. Sustainability.

[52] Liu F., Wang C., Zhang Y., Zhou S., Yang Y., Wu X., Hu F., Liu C.J. (2022). Data-driven analysis and evaluation of regional agriculture for high-quality development of Anhui Province in the Yangtze River Delta. Environ. Sci. Pollut. Res..

[53] Pan W., Wang J., Lu Z., Liu Y., Li Y. (2021). High-quality development in China: measurement system, spatial pattern, and improvement paths. Habitat Int..

[54] Jabareen Y. (2008). A new conceptual framework for sustainable development. Environ. Dev. Sustain..

[55] Mitlin D. (1992). Sustainable development: a guide to the literature. Environ. Urbanization.

[56] Ducarme F., Flipo F., Couvet D. (2021). How the diversity of human concepts of nature affects conservation of biodiversity. Conserv. Biol..

[57] Russell R., Guerry A.D., Balvanera P., Gould R.K., Basurto X., Chan K.M., Klain S., Levine J., Tam J. (2013). Humans and nature: how knowing and experiencing nature affect well-being. Annu. Rev. Environ. Resour..

[58] Welden E., Chausson A., Melanidis M.S. (2021). Leveraging Nature‐based Solutions for transformation: reconnecting people and nature. People Nat.

[59] Zingraff-Hamed A., Bonnefond M., Bonthoux S., Legay N., Greulich S., Robert A., Rotgé V., Serrano J., Cao Y., Bala R. (2021). Human–river encounter sites: looking for harmony between humans and nature in cities. Sustainability.

[60] Nathaniel S.P., Nwulu N., Bekun F. (2021). Natural resource, globalization, urbanization, human capital, and environmental degradation in Latin American and Caribbean countries. Environ. Sci. Pollut. Res..

[61] Yang M., Chen H., Long R., Wang Y., Hou C., Liu B. (2021). Will the public pay for green products? Based on analysis of the influencing factors for Chinese's public willingness to pay a price premium for green products. Environ. Sci. Pollut. Res..

[62] Sun C., Tong Y., Zou W. (2018). The evolution and a temporal-spatial difference analysis of green development in China. Sustain. Cities Soc..

[63] Sarkis J., Kouhizadeh M., Zhu Q.S. (2021). Digitalization and the greening of supply chains. Ind. Manag. Data Syst..

[64] Jiakui C., Abbas J., Najam H., Liu J., Abbas J. (2023). Green technological innovation, green finance, and financial development and their role in green total factor productivity: empirical insights from China. J. Clean. Prod..

[65] Zhang J., Chang Y., Zhang L., Li D. (2018). Do technological innovations promote urban green development?—a spatial econometric analysis of 105 cities in China. J. Clean. Prod..

[66] Eshetu G., Johansson T., Garedew W., Yisahak T. (2021). Determinants of smallholder farmers' adaptation options to climate change in a coffee-based farming system of Southwest Ethiopia. Clim. Dev..

[67] Dolan K.D., Mishra D.K. (2013). Parameter estimation in food science. Annu. Rev. Food Sci. Technol..

[68] Ren X., Tong Z., Sun X., Yan C. (2022). Dynamic impacts of energy consumption on economic growth in China: evidence from a non-parametric panel data model. Energy Econ..

[69] Huber F., Koop G., Onorante L., Pfarrhofer M., Schreiner J. (2023). Nowcasting in a pandemic using non-parametric mixed frequency VARs. J. Econom..

[70] Zhang Z., Zhang G., Su B. (2022). The spatial impacts of air pollution and socio-economic status on public health: empirical evidence from China. Soc. Econ. Plann. Sci..

[71] Autant‐Bernard C., LeSage J.P. (2011). Quantifying knowledge spillovers using spatial econometric models. J. Reg. Sci..

[72] Zhang M., Liu Y. (2022). Influence of digital finance and green technology innovation on China's carbon emission efficiency: empirical analysis based on spatial metrology. Sci. Total Environ..

[73] Huynh L.T.M., Gasparatos A., Su J., Dam Lam R., Grant E.I., Fukushi K. (2022). Linking the nonmaterial dimensions of human-nature relations and human well-being through cultural ecosystem services. Sci. Adv..

[74] Fang C., Wang Z., Liu H. (2020). Beautiful China Initiative: human-nature harmony theory, evaluation index system and application. J. Geogr. Sci..

[75] Lawson L.A., Nguyen-Van P. (2020). Is there a peaceful cohabitation between human and natural habitats? Assessing global patterns of species loss. Global Ecol. Conserv..

[76] Cai J., Li X., Liu L., Chen Y., Wang X., Lu S. (2021). Coupling and coordinated development of new urbanization and agro-ecological environment in China. Sci. Total Environ..

[77] He L., Shen J., Zhang Y. (2018). Ecological vulnerability assessment for ecological conservation and environmental management. J. Environ. Manag..

[78] Jiang H., Peng J., Zhao Y., Xu D., Dong J. (2022). Zoning for ecosystem restoration based on ecological network in mountainous region. Ecol. Indicat..

[79] Kang J., Li C., Li M., Zhang T., Zhang B. (2022). Identifying priority areas for conservation in the lower Yellow River basin from an ecological network perspective, Ecosyst. Health Sustainability.

[80] Zhang H., Wu D. (2023). The impact of rural industrial integration on agricultural green productivity based on the contract choice perspective of farmers. Agriculture.

[81] Kumar S., Raut R.D., Nayal K., Kraus S., Yadav V.S., Narkhede B.E. (2021). To identify industry 4.0 and circular economy adoption barriers in the agriculture supply chain by using ISM-ANP. J. Clean. Prod..

[82] Kamble S.S., Gunasekaran A., Gawankar S.A. (2020). Achieving sustainable performance in a data-driven agriculture supply chain: a review for research and applications. Int. J. Prod. Econ..

[83] Zhou Y., Xia Q., Zhang Z., Quan M., Li H. (2022). Artificial intelligence and machine learning for the green development of agriculture in the emerging manufacturing industry in the IoT platform. Ind. Manag. Data Syst..

[84] Szerement J., Szatanik-Kloc A., Jarosz R., Bajda T., Mierzwa-Hersztek M. (2021). Contemporary applications of natural and synthetic zeolites from fly ash in agriculture and environmental protection. J. Clean. Prod..

[85] Shafi M., Ramos-Meza C.S., Jain V., Salman A., Kamal M., Shabbir M.S., Rehman M.U. (2023). The dynamic relationship between green tax incentives and environmental protection. Environ. Sci. Pollut. Res..

[86] China's National Bureau of Statistics, China Statistical Yearbook. https://www.stats.gov.cn/sj/ndsj/(accessed on 8 March 2024).

[87] China Agriculture Database, China Rural Statistical Yearbook. https://vpn.gufe.edu.cn/https/77726476706e69737468656265737421fffb408c69357843700d9de29b5a2e7bdc1e/auth/platform.html?sid=6017DD05A9ED3F7851A85349276A6622_ipv442741116&cubeId=811 (accessed on 8 March 2024).

[88] China Agriculture and Forestry Database, China Agricultural Yearbook. https://vpn.gufe.edu.cn/https/77726476706e69737468656265737421fffb408c69357843700d9de29b5a2e7bdc1e/auth/platform.html?sid=6017DD05A9ED3F7851A85349276A6622_ipv442741116&cubeId=811 (accessed on 8 March 2024).

[89] China Green Food Development Center Annual Report of green food statistics. http://www.greenfood.agri.cn/ztzl/tjnb/lvsptjnb/.

[90] Guo C., Bai Z., Shi X., Chen X., Chadwick D., Strokal M., Zhang F., Ma L., Chen X. (2021). Challenges and strategies for agricultural green development in the Yangtze River Basin, J. Integr. Environ. Sci..

[91] Zhao Q., Pan Y., Xia X. (2020). Internet can do help in the reduction of pesticide use by farmers: evidence from rural China. Environ. Sci. Pollut. Res..

[92] Chi Y., Zhou W., Wang Z., Hu Y., Han X. (2021). The influence paths of agricultural mechanization on green agricultural development. Sustainability.

[93] Lei X., Shen Z.Y., Štreimikienė D., Baležentis T., Wang G., Mu Y. (2024). Digitalization and sustainable development: evidence from OECD countries. Appl. Energy.

[94] Arnaut J., Lidman J. (2021). Environmental sustainability and economic growth in Greenland: testing the environmental Kuznets curve. Sustainability.

